# Association Between Serum 25-Hydroxyvitamin D Status and Long-Term Marginal Bone Loss Around Dental Implants

**DOI:** 10.3390/jcm15197719

**Published:** 2026-10-07

**Authors:** Okan Özen, Vesile Elif Toy

**Affiliations:** Department of Periodontolgy, Faculty of Dentistry, Inonu University, 44280 Malatya, Turkey; elif.toy@inonu.edu.tr

**Keywords:** dental implants, marginal bone loss, osseointegration, vitamin D, 25-hydroxyvitamin D

## Abstract

**Background and Objectives:** The objective was to evaluate the association between serum 25-hydroxyvitamin D [25(OH)D] measured before functional loading and long-term peri-implant marginal bone loss (MBL). **Materials and Methods:** This retrospective study included 60 systemically healthy patients with 136 implants. Patients were classified as vitamin D-deficient (<20 ng/mL; *n* = 30) or -sufficient (≥20 ng/mL; *n* = 30). MBL was calculated from periapical radiographs obtained at prosthetic delivery and final follow-up (mean, 39.46 months; range, 34–48 months). Clinical parameters and implant loss were secondary outcomes. Between-group comparisons used the Mann–Whitney U and Fisher’s exact tests; associations were examined using Spearman’s rank correlation. **Results:** MBL was greater in the deficient group than in the sufficient group (0.98 ± 0.25 mm vs. 0.42 ± 0.32 mm; *p* < 0.001). Serum 25(OH)D was inversely correlated with MBL in the deficient group (r_s_ = −0.720), sufficient group (r_s_ = −0.604), and overall sample (r_s_ = −0.862; all *p* < 0.001). No statistically significant between-group differences were detected in the clinical parameters (all *p* > 0.05). **Conclusions:** Lower baseline serum 25(OH)D was associated with greater long-term MBL, while clinical parameters and patient-level implant loss did not differ between groups.

## 1. Introduction

Dental implant treatment is used to rehabilitate partially or completely edentulous patients and may improve oral health-related quality of life and perceived aesthetics [1]. Osseointegration, the direct structural and functional connection between living bone and the implant surface, is important for implant stability and long-term clinical performance [2]. The establishment and maintenance of this connection are influenced by several factors, including surgical technique, implant design, prosthetic loading conditions, bone quantity and quality, and patient-related biological characteristics [3]. Once functional loading begins, preservation of the peri-implant marginal bone becomes an important indicator of long-term peri-implant tissue stability [4].

Among the patient-related factors that may influence peri-implant bone, vitamin D has received increasing attention because of its role in skeletal metabolism. Vitamin D is a fat-soluble vitamin involved in calcium and phosphate homeostasis [5]. Its active metabolite acts through nuclear vitamin D receptors expressed in various cell types, and its biological functions extend beyond the skeletal system [6,7]. Low vitamin D status has been reported in different populations and clinical settings, making vitamin D deficiency an important public health concern [8,9,10,11].

Within bone tissue, vitamin D facilitates the intestinal absorption of calcium and phosphate and contributes to mineralization and remodelling through its effects on osteoblasts and osteoclasts [12,13]. These actions form part of a broader regulatory network involving parathyroid hormone, fibroblast growth factor 23, and Klotho [14]. At the peri-implant level, a systematic review reported that vitamin D deficiency was associated with reduced new bone formation and/or bone-to-implant contact (BIC) in experimental studies, whereas vitamin D supplementation generally increased BIC and/or new bone formation around implants [15]. A recent systematic review including both animal and human studies similarly reported that vitamin D deficiency was associated with impaired new bone formation and BIC, while most human studies supported an association between adequate vitamin D levels and improved implant osseointegration or reduced early implant failure [16].

Much of the current evidence concerning the relationship between vitamin D and implant osseointegration has been obtained from experimental animal studies [17,18,19]. Clinical studies remain limited, particularly those investigating long-term peri-implant marginal bone loss (MBL) in patients with vitamin D deficiency. The present study therefore aimed to compare peri-implant MBL after at least 34 months of functional loading between patients with deficient and sufficient serum 25-hydroxyvitamin D [25(OH)D] levels measured before functional loading and to examine the association between serum 25(OH)D concentrations and MBL. Peri-implant clinical parameters and implant survival were also evaluated as secondary outcomes. The study hypothesis was that patients with vitamin D deficiency would exhibit greater peri-implant MBL than those with sufficient vitamin D levels.

## 2. Materials and Methods

The study protocol was approved by the Inonu University Scientific Research Ethics Committee (2025-8698). The study was conducted using fully anonymized archived data in accordance with institutional and national requirements and with the principles of the Declaration of Helsinki. All patients had provided written informed consent for their implant treatment as part of routine clinical care. This treatment-related consent was distinct from consent for participation in the present retrospective study. During the preparation of this study, the authors used GenAI (ChatGPT, GPT-5.6, OpenAI) for the purposes of assisting with aspects of the study design, interpretation of the results, and language editing. The authors have reviewed and edited the output and take full responsibility for the content of this publication.

### 2.1. Study Design

This single-center retrospective cohort study evaluated the association between serum 25(OH)D levels measured before functional loading and subsequent peri-implant marginal bone loss. Archived clinical records and intraoral periapical radiographs of patients rehabilitated with fixed implant-supported prostheses were reviewed. The radiograph obtained at prosthetic delivery and functional loading was defined as baseline (T0), while the final follow-up radiograph taken at least 34 months after loading was defined as T1. The primary outcome was the change in peri-implant marginal bone level between T0 and T1. Peri-implant clinical parameters recorded at T1 and implant survival during follow-up were evaluated as secondary outcomes.

### 2.2. Patient Selection

**Figure 1:**
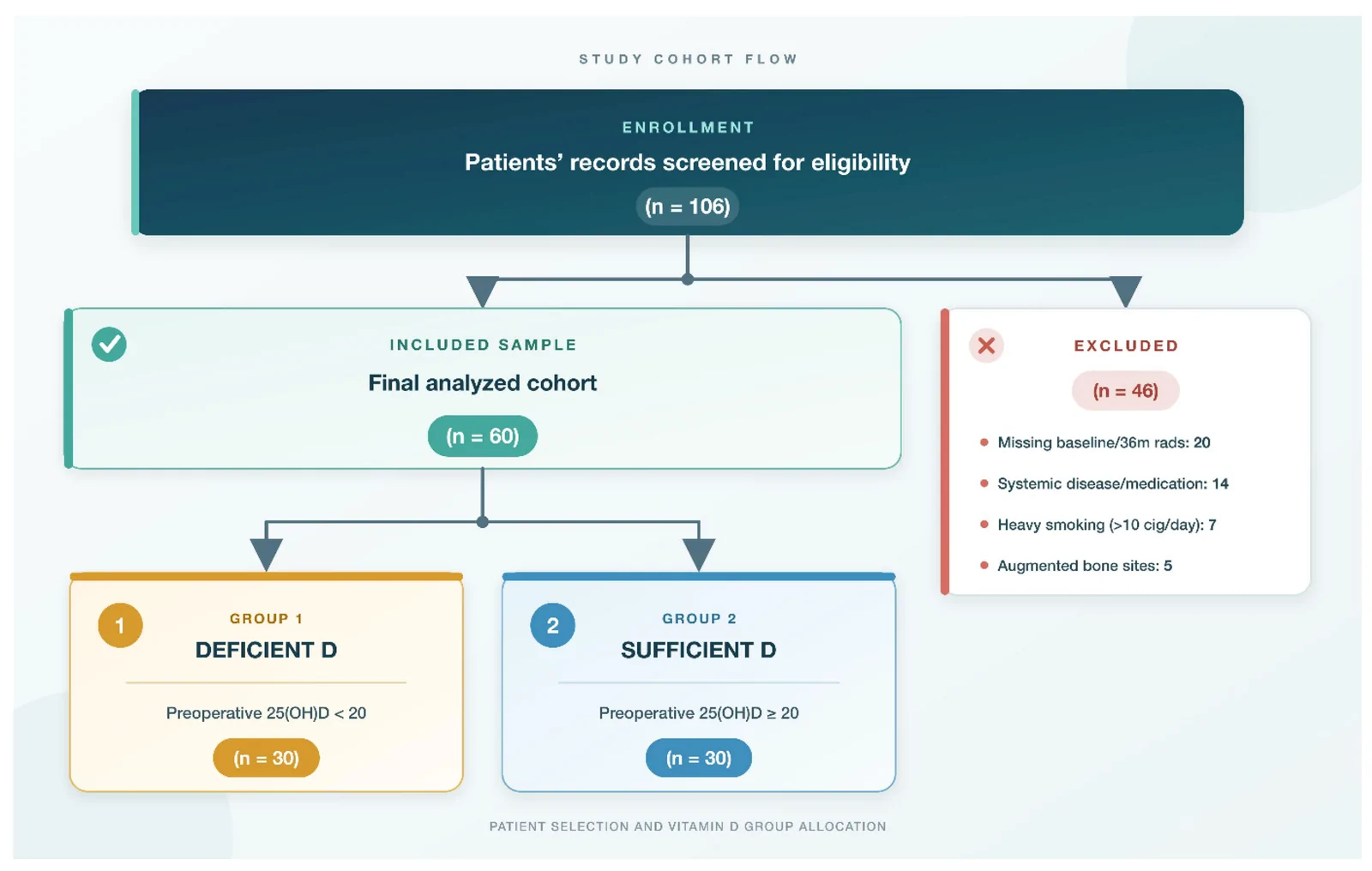
STROBE flow diagram detailing patient screening, eligibility assessment, specific exclusion criteria, final analyzed sample size (*n* = 60), and group allocation based on serum 25(OH)D status before functional loading (Group 1: Deficient [<20 ng/mL], Group 2: Sufficient [≥20 ng/mL]).

Based on the MBL data reported in a previous study [20], the sample size calculation indicated that at least 26 patients per group were required to detect an effect size of d = 0.80 with 80% power at a two-sided α level of 0.05, assuming a standard deviation of 0.6. The calculation was performed using G*Power version 3.1.9.4 (Franz Faul, Universität Kiel, Kiel, Germany). Patients with one or more dental implants restored with a fixed implant-supported prosthesis and in function for a minimum of 34 months were screened during routine follow-up visits at the Department of Periodontology, Inonu University Faculty of Dentistry. Eligibility was assessed by a periodontist (VET) through a review of medical and dental histories, surgical and prosthetic records, follow-up data, available radiographs, and relevant laboratory results. A total of 60 patients met the eligibility criteria and were included (Figure 1). Potential confounding factors included patient-related characteristics, implant-related factors, prosthetic characteristics, oral hygiene and peri-implant clinical status, and implant location. Some implant- and prosthesis-related variables were not available in the retrospective records and therefore could not be controlled.

Inclusion criteria:

1. Patients aged 18–60 years at the time of implant placement.
2. Receipt of at least one endosseous dental implant restored with a fixed implant-supported prosthesis, with clinical and radiographic follow-up available for at least 34 months after functional loading.
3. Availability of complete medical, dental, surgical, prosthetic, and follow-up records, including a serum 25(OH)D measurement obtained before functional loading.
4. Availability of standardized intraoral periapical radiographs obtained at prosthetic delivery and functional loading (T0) and at least 34 months after functional loading (T1).
5. Good oral hygiene at T0, defined as a full-mouth plaque score of <20% and a full-mouth bleeding score of <20%.

Exclusion criteria:

1. Systemic conditions affecting bone or vitamin D metabolism or peri-implant healing, including uncontrolled diabetes, untreated thyroid/parathyroid disorders, advanced renal or hepatic disease, malabsorption, and metabolic bone disease.
2. Previous head and neck radiotherapy or systemic chemotherapy.
3. Use of medications affecting bone turnover within 12 months before T0, including antiresorptive or antiangiogenic agents, long-term systemic corticosteroids, anabolic bone agents, and hormone replacement therapy.
4. Smoking more than 10 cigarettes per day at T0 or documented alcohol/substance use disorder.
5. Active untreated moderate-to-severe periodontitis at T0.
6. Implants placed in augmented bone sites (e.g., extensive guided bone regeneration with block grafts, autogenous bone grafting, or major sinus floor elevation procedures).
7. Bruxism or other documented parafunctional habits that could substantially affect occlusal loading.

At the final follow-up examination (T1), performed at least 34 months after functional loading, peri-implant clinical parameters were recorded to assess soft-tissue health and compare local plaque and inflammatory conditions between the vitamin D groups. For each implant remaining in function, plaque index (PI) [21], gingival index (GI) [22], bleeding on probing (BOP) [23], and probing depth (PD) were assessed at six sites: mid-buccal, mid-lingual, mesio-buccal, disto-buccal, disto-lingual, and mesio-lingual. BOP was expressed as the percentage of sites showing bleeding. Keratinized mucosa width (KMW) was measured at the mid-buccal aspect from the peri-implant mucosal margin to the mucogingival junction [24]. All measurements were made using a Williams periodontal probe (Hu-Friedy, Chicago, IL, USA), with PD and KMW recorded in millimetres. For patients with multiple implants, implant-level PI, GI, BOP, PD, and KMW values were averaged to obtain a single patient-level value for statistical analysis.

### 2.3. Data Collection and Patient Grouping

Data were extracted directly from patients’ records. In all patients, prosthetic delivery and functional loading were performed approximately three months after implant placement. Following the final periodontal evaluation and before functional loading, fasting venous blood samples were obtained for serum 25(OH)D assessment. Patients then proceeded to the prosthetic phase, with impressions taken on the same day as blood sampling. Patients were classified according to serum 25(OH)D levels as vitamin D-deficient (<20 ng/mL; Group 1, *n* = 30) or vitamin D-sufficient (≥20 ng/mL; Group 2, *n* = 30), using the 20 ng/mL threshold for vitamin D adequacy recommended by the Institute of Medicine [25]. All serum 25(OH)D measurements were obtained during the same seasonal period (April–May), thereby minimizing potential variability related to seasonal fluctuations in vitamin D status. All definitive prostheses were cement-retained metal-ceramic restorations. Patients with prosthetic complications during the follow-up period were excluded. For patients with multiple implants, the same loading protocol and prosthetic approach were applied to all included implants within the patient.

### 2.4. MBL Assessment

**Figure 2:**
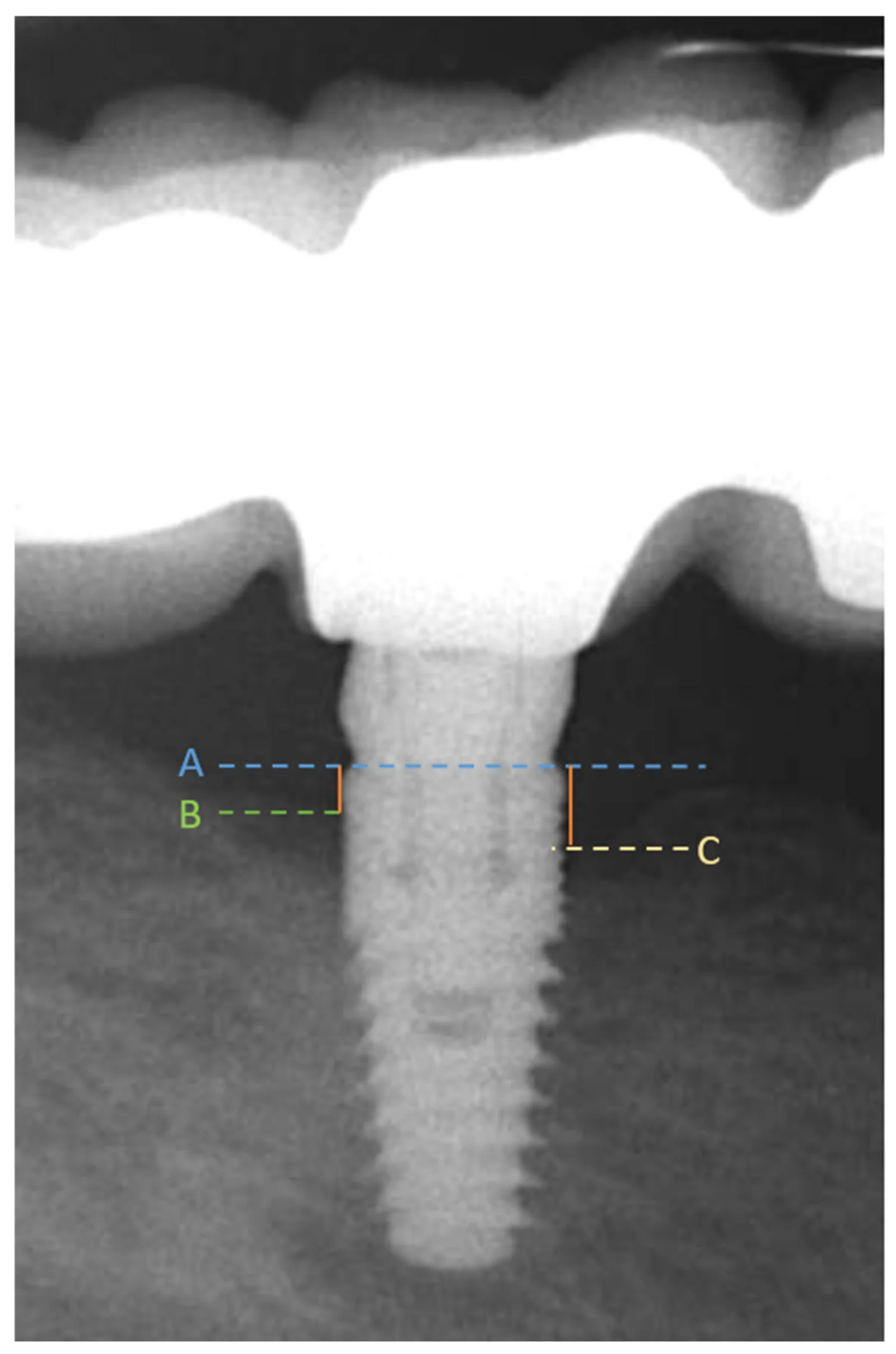
Periapical radiographic measurement of MBL. (A) The most coronal point of the intraosseous portion of the implant, used as the reference point. (B) The most apical point of the distal marginal bone defect. (C) The most apical point of the mesial marginal bone defect. Distal and mesial MBL were determined as the distances between A–B and A–C, respectively. MBL: marginal bone loss.

Peri-implant MBL was evaluated using intraoral periapical radiographs obtained at prosthetic delivery (T0) and at the final follow-up examination (T1), performed at least 34 months after functional loading. All radiographs were acquired using the paralleling technique according to a previously validated protocol [26]. Astandardized positioning system (Kerr X-ray Sensor Holder, Brea, CA, USA) was used at both T0 and T1. A photostimulable phosphor (PSP) plate system (Digora Optime, Soredex, Tuusula, Finland) was used as the image receptor. Radiographic images were acquired using a PHOT-X IIs intraoral X-ray unit (Takara Belmont Corporation, Osaka, Japan) at 70 kV, 6 mA, and an exposure time of 0.16 s. Projection-related distortion was minimized by using the same paralleling technique and positioning system at both time points. Radiographs of inadequate diagnostic quality, including those with projection errors or insufficient visualization of the implant platform and marginal bone, were excluded. Patients were excluded if either the T0 or T1 radiograph was unsuitable for MBL assessment. Accordingly, all implants included in the final analysis were assessable at both timepoints. Radiographic measurements were performed using ImageJ software (version 1.54g National Institutes of Health, Bethesda, MD, USA). Prior to radiographic measurements, image calibration was performed using an implant of known length as a reference to correct for radiographic magnification, and all measurements were recorded in millimeters. The distance from the implant platform to the most coronal bone-to-implant contact was measured at the mesial and distal aspects of each implant. MBL was calculated as the change in the implant platform-to-bone contact distance from T0 to T1 (T1–T0), with positive values indicating marginal bone loss. For each implant, the greater of the mesial and distal MBL values was selected as the implant-level MBL value. For patients with multiple implants, these implant-level MBL values were subsequently averaged to obtain a single patient-level MBL value. This approach was used because serum 25(OH)D status and the study groups were defined at the patient level and to avoid treating multiple implants within the same patient as statistically independent observations. As a sensitivity analysis, MBL was additionally recalculated using the mean of the mesial and distal measurements for each implant rather than the greater value. For patients with multiple implants, these implant-level mean MBL values were subsequently averaged to obtain a single patient-level value. The between-group comparison, correlation analysis, and adjusted multiple linear regression were then repeated using this alternative MBL definition. The measurement procedure is illustrated in Figure 2.

### 2.5. Examiner Reliability

All radiographic measurements were performed by a single examiner who was blinded to the patients’ serum 25(OH)D status and group allocation. Intra-examiner reliability was assessed by repeating the measurements on 20 randomly selected radiographs after a 2-week interval. During the repeated measurements, the examiner was blinded to the results of the initial measurements. Reliability was evaluated using a two-way random-effects model with absolute agreement and single measurements [ICC (2, 1)]. The analysis demonstrated excellent intra-examiner reliability, with an ICC of 0.921 (95% CI: 0.891–0.942).

### 2.6. Statistical Analyses

Statistical analyses were performed using IBM SPSS Statistics version 22.0 (IBM Corp., Armonk, NY, USA), while multiple linear regression analysis was performed using jamovi version 2.7.38 (The jamovi project, Sydney, Australia). The patient was considered the statistical unit of analysis. Accordingly, implant-level observations were not treated as independent data points in group comparisons, correlation analyses, or regression models. Comparisons of MBL between the vitamin D groups and correlation analyses between serum 25(OH)D levels and MBL were therefore performed using patient-level MBL values (*n* = 60). Continuous variables were summarized as mean, standard deviation, median, minimum, and maximum, while categorical variables were presented as frequencies and percentages. Distributional characteristics of continuous variables were evaluated using descriptive statistics, graphical inspection, and the Kolmogorov–Smirnov test. For variables that did not meet the normality assumption, between-group comparisons were performed using the Mann–Whitney U test. For the primary between-group comparison of MBL, the estimated between-group difference with its 95% confidence interval (CI) and rank-biserial correlation as an effect-size measure were additionally reported. Categorical variables were analyzed using Fisher’s exact test, the Fisher–Freeman–Halton exact test, or Yates’ continuity correction, as appropriate. Spearman’s rank correlation analysis was used to assess the relationships between serum 25(OH)D levels and PI, GI, BOP, PD, KMW, and MBL. Statistical significance was set at *p* < 0.05. No formal adjustment for multiple comparisons was applied. Therefore, analyses involving multiple secondary clinical parameters and correlation analyses were considered exploratory and interpreted with consideration of the potential inflation of type I error. A multiple linear regression analysis was performed to evaluate the association between serum 25(OH)D levels and MBL after adjustment for available potential confounding factors. MBL was entered as the dependent variable, while serum 25(OH)D level, age, sex, follow-up duration, PI, PD, and KMW were included as independent variables. Regression coefficients (B) with 95% confidence intervals (CIs) were reported. To facilitate clinical interpretation, the regression coefficient for serum 25(OH)D was additionally expressed per 10 ng/mL increase, with the corresponding 95% CI. Multicollinearity was assessed using variance inflation factors (VIFs), and regression assumptions were evaluated using residual diagnostics. A sensitivity analysis was performed using the mean mesial/distal MBL as an alternative outcome, with the same patient-level analytical approach and covariate adjustment as in the primary analysis.

## 3. Results

### 3.1. Demographic and Baseline Characteristics

A total of 60 systemically healthy patients, including 23 men (38.3%) and 37 women (61.7%), were evaluated. The mean age was 50.13 ± 6.28 years (range, 39–60 years). The groups did not differ significantly in age (*p* = 0.415) or sex distribution (*p* = 1.000). Serum 25(OH)D levels were 9.03 ± 1.77 ng/mL (median, 9.2 ng/mL; range, 4.6–12.0 ng/mL) in Group 1 and 34.28 ± 14.11 ng/mL (median, 29.7 ng/mL; range, 21.5–89.3 ng/mL) in Group 2 (*p* = 0.001) (Table 1).

### 3.2. Follow-Up Duration and Implant Characteristics

A total of 136 implants were evaluated, including 67 implants in Group 1 and 69 implants in Group 2. The mean follow-up duration was 39.23 ± 3.22 months in Group 1 (range, 36–48 months) and 39.70 ± 4.05 months in Group 2 (range, 34–48 months), with no significant between-group difference (*p* = 0.792). The groups also showed no significant differences in the number of implants per patient (*p* = 1.000) or their distribution by jaw (*p* = 0.643) (Table 2).

### 3.3. Clinical Parameters and Peri-Implant MBL

**Figure 3:**
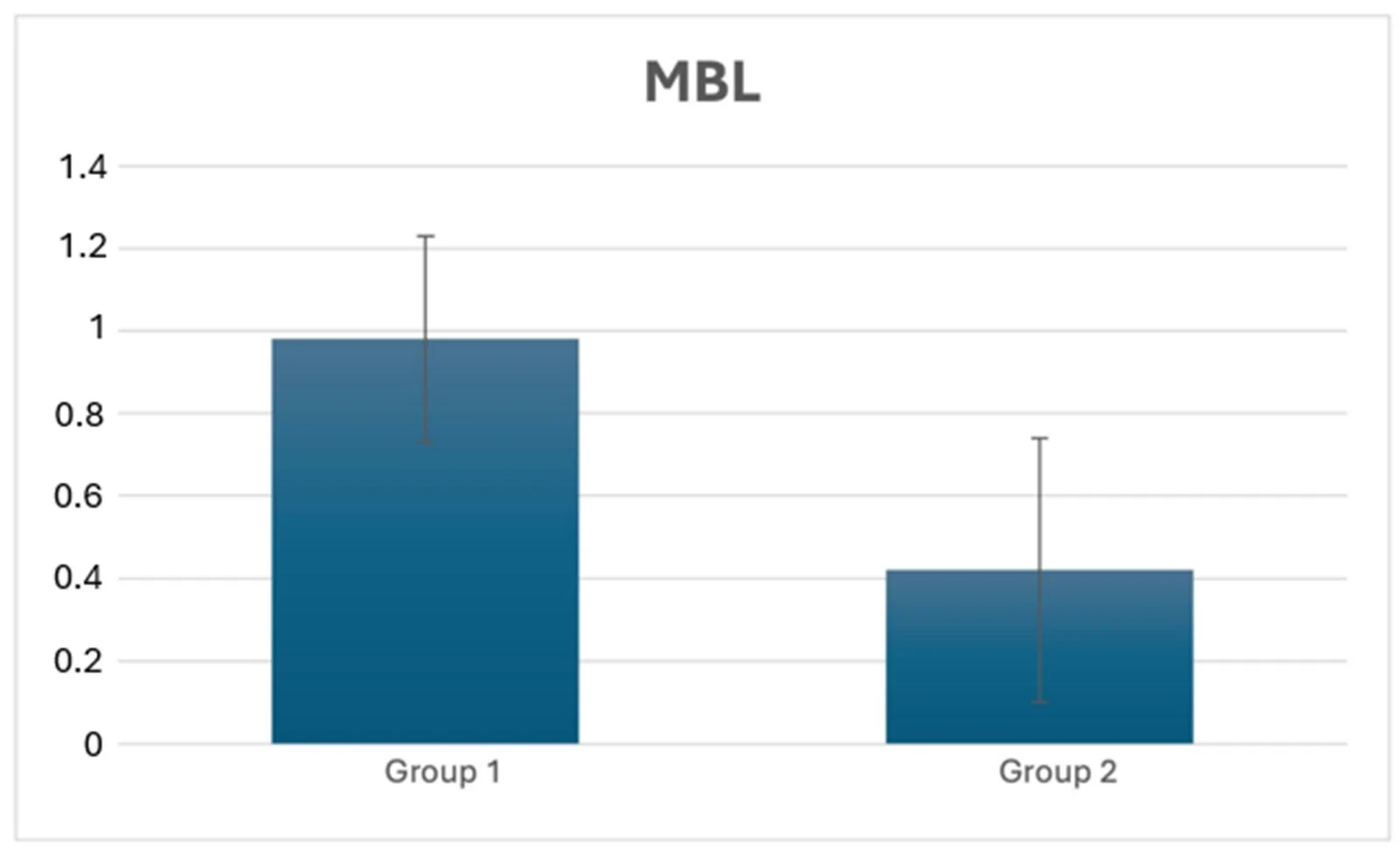
Comparison of peri-implant MBL at the final follow-up between Group 1 and Group 2. MBL: marginal bone loss.

No significant differences were observed between Group 1 and Group 2 in PI (*p* = 0.428), GI (*p* = 0.835), BOP (*p* = 0.545), PD (*p* = 0.068), or KMW (*p* = 0.159). In contrast, at the patient level, MBL was significantly greater in Group 1 than in Group 2 after a mean follow-up of 39.46 months (0.98 ± 0.25 mm vs. 0.42 ± 0.32 mm; Mann–Whitney U = 66.5, *p* < 0.001). The estimated between-group difference was 0.55 mm (95% CI: 0.35–0.70 mm), with a rank-biserial correlation of −0.852 (Table 3, Figure 3). In an exploratory descriptive analysis, implant loss occurred in 3 of 30 patients in Group 1 (10.0%; 95% CI: 2.1–26.5%) and in 0 of 30 patients in Group 2 (0%; 95% CI: 0–11.6%). Given the small number of events, no inference regarding implant survival was made (Table 3).

In the multiple linear regression analysis, serum 25(OH)D level remained inversely associated with MBL after adjustment for age, sex, follow-up duration, PI, PD, and KMW (B = −0.0176 mm/ng/mL, 95% CI: −0.0222 to −0.0129, *p* < 0.001). Expressed per 10 ng/mL increase, higher serum 25(OH)D concentration was associated with 0.176 mm lower MBL (B = −0.176 mm per 10 ng/mL, 95% CI: −0.222 to −0.129 mm, *p* < 0.001). The model explained 57.8% of the variance in MBL (R^2^ = 0.578). No evidence of multicollinearity was observed (VIF range: 1.06–1.23).

In the sensitivity analysis using the mean of the mesial and distal MBL measurements, the between-group difference remained statistically significant (Mann–Whitney U = 130, *p* < 0.001). Serum 25(OH)D remained inversely correlated with MBL (r_s_ = −0.402, *p* = 0.001). In the adjusted multiple linear regression analysis, serum 25(OH)D also remained inversely associated with MBL (B = −0.00735 mm/ng/mL, 95% CI: −0.01199 to −0.00272, *p* = 0.002), supporting the robustness of the primary findings.

### 3.4. Correlation Analysis

Spearman’s rank correlation analysis showed no significant correlations between serum 25(OH)D levels measured before functional loading and the evaluated clinical parameters (PI, GI, BOP, PD, and KMW) in Group 1, Group 2, or the overall study population (*p* > 0.05).

**Figure 4:**
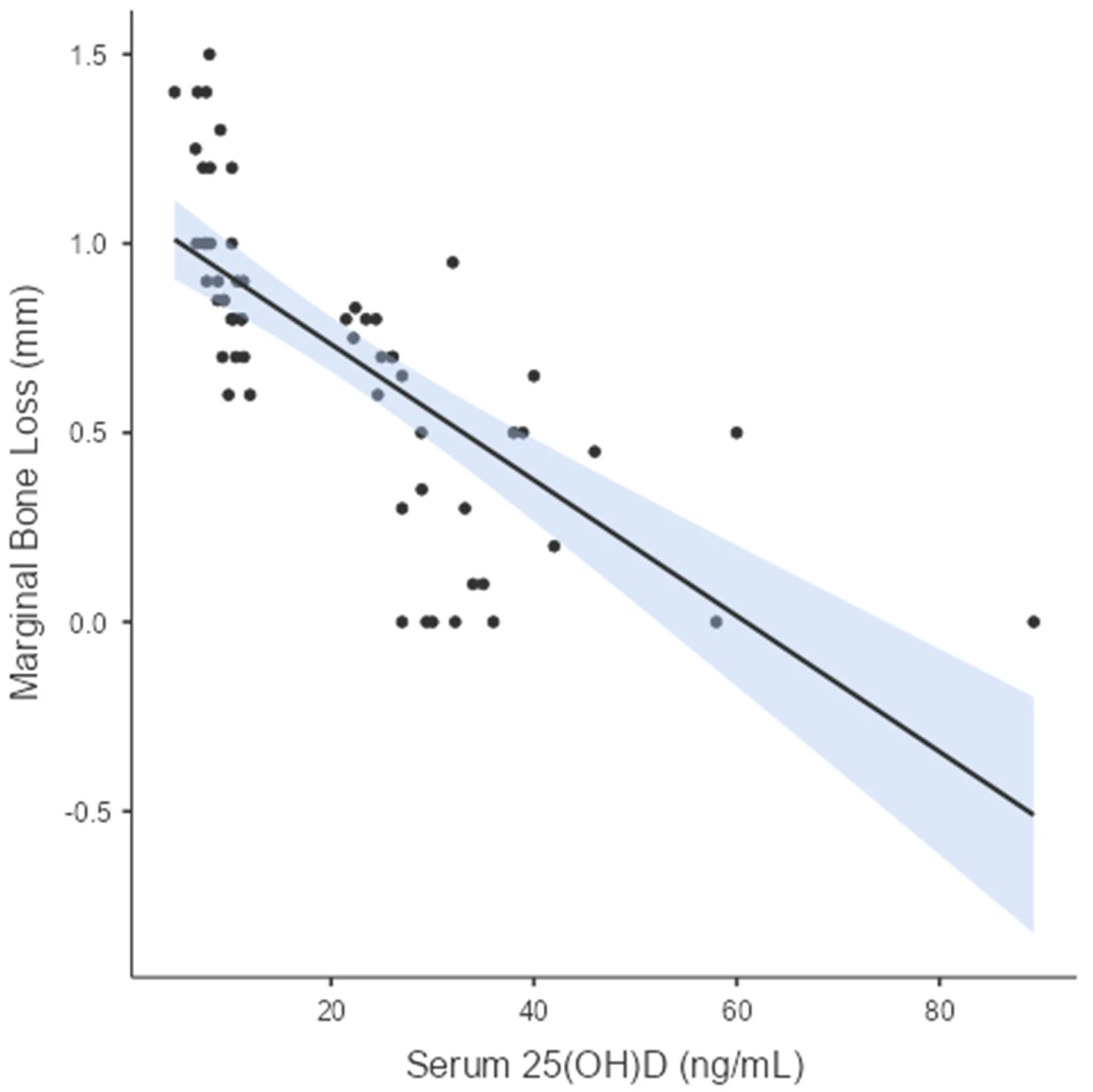
Scatter plot showing the relationship between individual serum 25(OH)D concentrations and patient-level marginal bone loss (MBL) in the overall study population. The solid line represents the fitted linear regression line, and the shaded area represents the 95% confidence interval.

Serum 25(OH)D levels were inversely correlated with peri-implant MBL in Group 1 (r_s_ = −0.720; *p* < 0.001), Group 2 (r_s_ = −0.604; *p* < 0.001), and the overall study population (r_s_ = −0.862; *p* < 0.001) (Table 4, Figure 4).

## 4. Discussion

Vitamin D plays an important role in bone metabolism [27] and may therefore contribute to the maintenance of peri-implant bone after functional loading. The main finding of the present study was that patients with deficient serum 25(OH)D levels had 0.56 mm greater MBL than patients with sufficient levels after a mean follow-up of 39.46 months. In addition, serum 25(OH)D levels were inversely correlated with MBL, while peri-implant clinical indices were similar between the groups. Although implant loss occurred only in the vitamin D-deficient group, the difference between the groups was not statistically significant. These findings indicate an association between lower serum 25(OH)D levels and greater peri-implant MBL. Although vitamin D has an established role in bone metabolism and may provide a biologically plausible explanation for this relationship, the observational design of the present study does not allow causal inference. Importantly, the main findings remained statistically significant in the sensitivity analysis using the mean of the mesial and distal MBL measurements, supporting the robustness of the observed association.

Although the between-group difference in MBL was statistically significant, its clinical relevance should be interpreted cautiously. The observed difference of approximately 0.56 mm should be considered in the context of physiological peri-implant bone remodeling and the magnitude of marginal bone level changes reported during long-term functional follow-up. Previous meta-analytic data have shown mean marginal bone-level changes of approximately 0.56–0.68 mm during 2–5 years of follow-up, depending on implant location [28]. A recent meta-analysis also reported a mean MBL of 0.79 mm (95% CI: 0.32–1.26 mm) after 5 years of loading for implants placed in pristine bone [29]. Importantly, peri-implantitis is not defined by a single threshold of radiographic bone loss alone; current consensus criteria emphasize progressive bone loss together with clinical signs of peri-implant inflammation [30]. Therefore, although the greater MBL observed in the vitamin D-deficient group may indicate a less favorable peri-implant bone response, the 0.56 mm between-group difference should not by itself be interpreted as evidence of clinically relevant peri-implant disease.

The inverse relationship observed in the present study is consistent with the findings of Tabrizi et al. [31] who prospectively evaluated MBL during the first year after functional loading. They reported greater MBL in patients with lower mean serum 25(OH)D levels, whereas probing depth was similar between the groups. Similarly, in the present study, patients with vitamin D deficiency showed greater MBL despite having peri-implant clinical indices similar to those of patients with sufficient vitamin D levels. Although the vitamin D cutoff values and assessment periods differed between the studies, these findings suggest that radiographic changes associated with vitamin D status may occur without clear differences in clinical parameters. A more recent clinical study [32] also reported that lower preoperative 25(OH)D levels were associated with greater early crestal bone loss. However, peri-implant soft tissue thickness was also found to affect bone loss, highlighting the multifactorial nature of MBL.

The biological effects of vitamin D on bone metabolism and healing may provide a possible explanation for these clinical observations; however, the present study did not investigate biological mechanisms, and the observed statistical association should not be interpreted as evidence of a mechanistic relationship. Javed et al. [33] reviewed six animal studies and reported that five showed increased new bone formation and/or bone-to-implant contact after vitamin D_3_ supplementation. However, they also emphasized that the available evidence was insufficient to draw firm conclusions. Similarly, low vitamin D intake has been suggested as a possible factor that may negatively affect bone healing, and osteocytes and immune cells have been reported to play important regulatory roles in osseointegration and the maintenance of peri-implant bone [4]. In a preclinical study involving ovariectomized rats [18], vitamin D deficiency reduced cortical bone-to-implant contact four weeks after implant placement. However, similar effects were not observed for medullary or periosteal bone-to-implant contact, peri-implant bone area, or mineral apposition rate. Therefore, although preclinical findings provide biological support for a possible relationship between vitamin D and peri-implant bone response, they should not be directly generalized to long-term clinical outcomes.

Clinical studies evaluating the early healing period also provide some support for this biological relationship. In the prospective study by Kwiatek et al. [34], among individuals with serum 25(OH)D levels below 30 ng/mL, those who received vitamin D supplementation showed more favorable radiographic bone level changes at 12 weeks than those who did not receive supplementation. Similarly, another prospective study [35] reported that higher preoperative vitamin D levels were associated with greater implant stability at the third and sixth months. These studies mainly focused on early osseointegration, whereas the mean follow-up period of 39.46 months in the present study provides additional information on the longer-term behavior of peri-implant bone after functional loading.

The evidence regarding the relationship between vitamin D levels and implant loss is less consistent than that regarding MBL. Mangano et al. [36] observed increasing rates of early implant failure as preoperative serum vitamin D levels decreased, although the differences between the groups did not reach statistical significance. In the present study, implant loss occurred in three patients in Group 1 and in none in Group 2. However, given the very small number of events, this finding should be considered exploratory and does not allow reliable inference regarding an association between vitamin D status and implant survival. Moreover, Mangano et al. evaluated early implant failures occurring within the first four months after implant placement and before prosthetic restoration, whereas the present study evaluated MBL and implant loss during long-term functional follow-up. Therefore, direct comparisons should be made with caution.

More recent prospective studies have also reported conflicting findings regarding implant failure. One study [37] found a markedly higher rate of early implant failure in patients with severe vitamin D deficiency, supporting the trend reported by Mangano et al. [36]. However, the small number of patients in the severe deficiency subgroup should be considered when interpreting these findings. In contrast, Grigoraș et al. [38] found no statistically significant association between preoperative vitamin D levels and implant loss during the first year. Differences in vitamin D cutoff values, sample sizes, definitions of implant failure, follow-up periods, and control of potential confounding factors may partly explain these inconsistent results. Therefore, the relationship between vitamin D levels and implant failure appears to be less clear than the relationship with peri-implant bone level changes.

This variability in the clinical evidence is also reflected in recent reviews. A systematic review [39] reported a tendency toward less favorable MBL outcomes in patients with low serum vitamin D levels. However, only four heterogeneous studies were included, limiting the strength of the available evidence. In a more recent scoping review, Sodnom-Ish et al. [40] suggested that sufficient vitamin D levels may be associated with greater implant stability and lower rates of early implant failure and MBL. However, the authors emphasized the methodological differences among the available studies and the limited amount of long-term clinical data.

In this context, the present study contributes to the available literature by evaluating the relationship between serum 25(OH)D levels and peri-implant MBL over a mean functional follow-up period of more than three years. However, the observed association should not be interpreted as evidence of causality. Residual confounding and selection bias cannot be excluded because of the retrospective design. Vitamin D deficiency may also represent a marker of unmeasured patient characteristics rather than an independent determinant of MBL. In addition, the observational design does not allow the direction of the observed association to be established, and reverse causation cannot be completely excluded. Peri-implant bone loss may be influenced by several factors, including local anatomical characteristics, soft tissue phenotype, oral hygiene, prosthetic and occlusal factors, implant-related characteristics, and systemic variables. In addition, a single serum 25(OH)D measurement before functional loading may not fully represent the patients’ long-term vitamin D status. Therefore, long-term prospective studies with repeated serum 25(OH)D measurements, adequate control of relevant local and systemic confounding factors, and standardized radiographic follow-up protocols are needed to further clarify the relationship between vitamin D status and peri-implant bone maintenance and the clinical relevance of the observed 0.56 mm difference.

The present study has several limitations. First, despite adjustment for available potential confounders, its retrospective observational design does not allow causal inference, and residual confounding cannot be excluded. Selection bias is possible because inclusion depended on the availability of serum 25(OH)D results and radiographs suitable for MBL measurement at both time points; the direction of any resulting bias cannot be determined. Averaging implant-level measurements within each patient avoided treating multiple implants from the same patient as independent observations, but did not capture within-patient implant-level variability. No formal correction for multiple comparisons was applied to the secondary analyses, which may have increased the risk of type I error. Second, vitamin D status was determined using a single serum 25(OH)D measurement before functional loading. Changes in vitamin D status during follow-up could not be assessed, and information on vitamin D supplementation during this period was unavailable. Third, detailed information on implant manufacturer/system, surface characteristics, diameter, length, and position relative to adjacent anatomical structures was not consistently available. Prostheses were not analyzed according to single- versus multiple-unit configuration or occlusal scheme. These unmeasured implant- and prosthesis-related factors may have influenced MBL. The small number of implant failures limited their statistical evaluation. Finally, the single-center setting may limit the generalizability of the findings. Future prospective, multicenter studies could reduce these limitations by obtaining repeated serum 25(OH)D measurements, documenting supplementation and implant- and prosthesis-related factors, and using standardized radiographic follow-up. Implant-level analyses could account for multiple implants within the same patient. The observed association between serum 25(OH)D levels and peri-implant MBL should be interpreted in light of these limitations.

## 5. Conclusions

The findings of the present study suggest that lower serum 25(OH)D levels before functional loading may be associated with greater peri-implant marginal bone loss during long-term functional follow-up. Patients with vitamin D deficiency showed greater MBL than those with sufficient vitamin D levels despite similar peri-implant clinical parameters. Given the observational design and the potential for residual confounding, these findings should not be interpreted as evidence of a causal relationship. Prospective studies with repeated serum 25(OH)D measurements and adequate control of relevant confounding factors are needed to further evaluate this association.

## Figures and Tables

**Table 1:** Demographic characteristics and serum 25(OH)D levels according to vitamin D status.

Parameter	Group 1 (*n* = 30)	Group 2 (*n* = 30)	*p*-Value
Age (years)			
Mean ± SD	50.77 ± 6.16	49.5 ± 6.45	0.415 ^a^
Median (Min–Max)	53 (39–60)	49.5 (39–60)	
Serum 25(OH)D (ng/mL)			
Mean ± SD	9.03 ± 1.77	34.28 ± 14.11	0.001 ^a^
Median (Min–Max)	9.2 (4.6–12.0)	29.7 (21.5–89.3)	
Sex *n* (%)			
Male	12 (40%)	11 (36.7%)	1.000 ^b^
Female	18 (60%)	19 (63.3%)	

SD: standard deviation; 25(OH)D: 25-hydroxyvitamin D. ^a^ Mann–Whitney U test; ^b^ Yates’ continuity correction. The patient was the statistical unit of analysis (*n* = 30 per group).

**Table 2:** Follow-up duration and patient-level implant characteristics according to vitamin D status.

Parameter	Group 1 (*n* = 30)	Group 2 (*n* = 30)	*p*-Value
Observation period (months)			
Mean ± SD	39.23 ± 3.22	39.70 ± 4.05	0.792 ^a^
Median (Min–Max)	39.00 (36.00–48.00)	38.50 (34.00–48.00)	
Number of implants, *n* (%)			
1 implant	4 (13.3%)	3 (10.0%)	1.000 ^b^
2 implants	19 (63.3%)	20 (66.7%)	
3 implants	3 (10.0%)	2 (6.7%)	
4 implants	4 (13.3%)	5 (16.7%)	
Jaw location, *n* (%)			
Maxilla	9 (30.0%)	7 (23.3%)	0.643 ^b^
Mandible	20 (66.7%)	20 (66.7%)	
Both (Maxilla + Mandible)	1 (3.3%)	3 (10.0%)	

SD: standard deviation. ^a^ Mann–Whitney U test; ^b^ Fisher–Freeman–Halton exact test. The patient was the statistical unit of analysis (*n* = 30 per group). Implant number and jaw location represent patient-level characteristics.

**Table 3:** Patient-level comparison of peri-implant clinical parameters, marginal bone loss, and implant loss between the study groups.

Parameter	Group 1	Group 2	*p*-Value
PI			
Mean ± SD	0.61 ± 0.57	0.74 ± 0.66	0.428 ^a^
Median (Min–Max)	0.50 (0.00–2.00)	0.60 (0.00–2.40)	
GI			
Mean ± SD	0.91 ± 0.61	0.90 ± 0.70	0.835 ^a^
Median (Min–Max)	1.00 (0.00–2.00)	1.00 (0.00–2.00)	
BOP (%)			
Mean ± SD	34.42 ± 21.85	31.08 ± 24.25	0.545 ^a^
Median (Min–Max)	33.30 (0.00–66.60)	33.30 (0.00–66.60)	
PD (mm)			
Mean ± SD	2.96 ± 0.40	2.59 ± 0.94	0.068 ^a^
Median (Min–Max)	3.00 (2.50–3.70)	2.50 (1.20–5.00)	
KMW (mm)			
Mean ± SD	2.84 ± 1.09	3.38 ± 1.79	0.159 ^a^
Median (Min–Max)	2.60 (1.30–6.00)	3.40 (0.00–6.60)	
MBL (mm)			
Mean ± SD	0.98 ± 0.25	0.42 ± 0.32	<0.001 ^a^
Median (Min–Max)	0.90 (0.60–1.50)	0.50 (0.00–1.00)	
Estimated between-group difference (95% CI)	0.55 mm (0.35–0.70)		
Effect size (rank-biserial correlation, r_rb_)	−0.852		
Implant loss, *n* (%)			
Absent	27 (90.0%)	30 (100.0%)	0.237 ^b^
Present	3 (10.0%)	0 (0.0%)	

PI: plaque index; GI: gingival index; BOP: bleeding on probing; PD: probing depth; KMW: keratinized mucosal width; MBL: marginal bone loss; SD: standard deviation; CI: confidence interval; r_rb_: rank-biserial correlation. ^a^ Mann–Whitney U test; ^b^ Fisher’s exact test. The patient was the statistical unit of analysis (*n* = 30 per group). For patients with multiple implants, implant-level PI, GI, BOP, PD, KMW, and MBL values were averaged to obtain a single patient-level value. For the primary MBL comparison, r_rb_ was reported as the effect-size measure. Implant loss was evaluated at the patient level and should be considered an exploratory descriptive outcome because of the small number of events.

**Table 4:** Correlations between serum 25(OH)D levels and peri-implant clinical and radiographic parameters.

Parameter	Group 1 r_s_ (*p*-Value)	Group 2 r_s_ (*p*-Value)	Total Cohort r_s_ (*p*-Value)
PI	0.006 (0.976)	−0.126 (0.508)	0.066 (0.615)
GI	−0.172 (0.363)	0.159 (0.400)	−0.028 (0.833)
BOP	−0.174 (0.358)	0.168 (0.376)	−0.075 (0.570)
PD	−0.309 (0.097)	0.146 (0.440)	−0.227 (0.081)
KMW	0.086 (0.652)	−0.073 (0.700)	0.167 (0.201)
MBL	−0.720 (<0.001)	−0.604 (<0.001)	−0.862 (<0.001)

25(OH)D: 25-hydroxyvitamin D; PI: plaque index; GI: gingival index; BOP: bleeding on probing; PD: probing depth; KMW: keratinized mucosal width; MBL: marginal bone loss; r_s_: Spearman’s rank correlation coefficient. The patient was the statistical unit of analysis (*n* = 30 per group; total *n* = 60). For patients with multiple implants, implant-level PI, GI, BOP, PD, KMW, and MBL values were averaged to obtain a single patient-level value.

## Data Availability

The data presented in this study are available from the corresponding author upon reasonable request. The data are not publicly available due to patient privacy and ethical considerations.
